# Using an interprofessional competency framework to enhance collaborative pediatric nursing education and practice

**DOI:** 10.1186/s12912-022-00932-z

**Published:** 2022-06-10

**Authors:** Jill M. G. Bally, Shelley Spurr, Shannon Hyslop, Heather Hodgson-Viden, Erick D. McNair

**Affiliations:** 1 College of Nursing, University of Saskatchewan, 104 Clinic Place, Saskatoon, SK S7N 2Z4 Canada; 2 College of Medicine, University of Saskatchewan, 107 Wiggins Road, Saskatoon, SK S7N 5E5 Canada

**Keywords:** Interprofessional education, Interprofessional collaboration, Pediatrics, Nursing education, Quantitative research, Interprofessional competency framework

## Abstract

**Background:**

Interprofessional education (IPE) provides healthcare students with the knowledge and skills necessary to provide safe and effective collaborative care in a variety of clinical settings. Inclusion of IPE in nursing curricula is required for program accreditation in Canada; a variety of learning strategies at varied levels are used to meet this requirement. As this formal requirement only occurred over the last decade, development, facilitation, and evaluation of IPE interventions are ongoing.

**Purpose:**

The purpose of this study was to examine if exposure to an introductory IPE activity influenced third-year undergraduate nursing students’ perceived ability to practice competent interprofessional collaboration (IPC).

**Methods:**

The introductory IPE activity included ten-hours of interactive lectures and related case studies, grounded in the National Interprofessional Competency Framework, delivered by various healthcare professionals in a third-year nursing theory and clinical course. Following completion of the courses, quantitative data were collected via the Interprofessional Collaborative Competencies Attainment Survey (ICCAS) which was used to evaluate nursing students’ change in competencies for IPC. Frequencies, percentages, and means were used to analyze the demographic data, the Cronbach’s alpha coefficient was used to evaluate the internal reliability of the ICCAS, and paired t-tests were conducted to measure the difference from pre- to post-participation for all 20 items and 6 subscales of the ICCAS.

**Results:**

Study participants (*n* = 111) completed the ICCAS at the end of the courses to measure change in six competencies. The survey results indicated improvements in all competencies following the IPE activity.

**Conclusions:**

The significant findings demonstrate that exposure to introductory IPE activities, involving nursing students and other healthcare professionals, hold promise for enhancing IPC in pediatric clinical settings. These findings can be used to inform the development of formal IPE interventions.

Interprofessional education (IPE) provides students in healthcare professions with knowledge and skills to engage in interprofessional collaboration (IPC) [1]. Interprofessional collaboration among healthcare professionals is essential to improving the quality and accessibility of healthcare [1]. As such, IPE plays an important role in the development of future healthcare professionals, including nurses [2, 3]. Nurse educators have been called on to transform nursing education including curricular content and clinical opportunities, to prepare nursing students to participate in IPC, the new paradigm of health care delivery [4].

A variety of learning strategies have been used to integrate IPE into pre-licensure nursing curricula across North America [5] including simulation [6–10], case studies [11, 12], IPE-focused courses [12, 13], virtual patient cases [14], clinical experiences in community-based settings [15, 16], and combinations of two or more of the above [17, 18]. However, a formal requirement for IPE in nursing curricula in Canada was only introduced in the last decade, when it became a part of program accreditation [19]. As such, many institutions, particularly in Canada [20], remain in the early stages of incorporating IPE into curricula [21]. In addition, children and their families are less commonly the focus of IPE in healthcare professional education; calls have been made for these priorities to be changed in countries such as Canada [22]. However, research gaps remain surrounding which methods are most effective for implementing IPE, particularly as comparable research designs are lacking [21, 23], and the extent to which IPC goals are being met with various IPE activities [24]. Nurse educators continue to be called on to create and evaluate innovative IPE activities that enhance the ability of nursing students to participate in IPC [13].

The Canadian Interprofessional Health Collaborative (CIHC) [25], made up of healthcare educators, researchers, students, and professionals across the nation, created the National Interprofessional Competency Framework to provide a set of competencies that are at the foundation of effective IPC, informed by literature, and measurable. The Framework describes six competencies that are essential for IPC: [1] *role clarification*, [2] *patient/client/family/community-centered care*, [3] *team functioning*, [4] *collaborative leadership*, [5] *interprofessional conflict resolution*, and [6] *interprofessional communication* [25]. The competencies outlined in the National Interprofessional Competency Framework were used to develop the Interprofessional Collaborative Competency Attainment Survey [ICCAS] [26], which is an IPE assessment instrument that includes six subscales that align with those included in the Framework [27]. Thus, the ICCAS [26] may be a suitable way to understand the extent to which IPC goals are being met with IPE activities, and provide comparable research results across various interventions.

Recognizing that IPE is a crucial aspect of healthcare professional education, the University of Saskatchewan developed an administrative leadership team called the Council of Health Sciences Deans (CHSD) which include the Colleges of Dentistry, Kinesiology, Medicine, Nursing, Pharmacy and Nutrition, Veterinary Medicine, and Schools of Physical Therapy and Public Health. The vision and mission of the CHSD is that the health sciences at this University be leaders in advancing health, locally and globally, through excellence in IPE and practice. To meet the demands of the nursing program accreditation and to work within the mission of the University, the College of Nursing Bachelor of Science in Nursing (BSN) program adopted a curriculum model that was based on several key principles including IPE. As such, IPE has been embedded in the curriculum and identified as the learning outcomes of specific courses to ensure the progression of this theme throughout the curriculum. In one particular course in the BSN program, nurse educators integrated an introductory IPE activity into a nursing theory course. This IPE activity was based on the six competencies outlined in the National Interprofessional Competency Framework [25] and included faculty from the Colleges of Nursing, Medicine (a perfusionist, pediatric endocrinologist, pediatric palliative care specialist, and pediatric oncologist), and Dentistry (a pediatric Public Health Dentist) to create a learning environment for nursing students that helps to promote IPC in pediatric practice. The introductory IPE activity provided exposure to IPE and reinforced an understanding that there are many healthcare professionals with unique and shared bodies of knowledge and skills [28]. The effectiveness of this IPE activity has not previously been tested. Thus, the purpose of this study was to examine if exposure to this introductory IPE activity led to a change in the perceived ability of third-year undergraduate nursing students to practice competent IPC. The research question guiding this study included the following: Does the inclusion of an interprofessional teaching team and a focus on the National Interprofessional Competency Framework in a pediatric theory and clinical course influence the perceived ability of nursing students to engage in IPC?

## Background

The Centre for the Advancement of Interprofessional Education (CAIPE) [29] provides a well-accepted definition for IPE: “When two or more professions learn about, with and from each other, to improve collaborative practice and the quality of care. The introductory IPE activity evaluated in this paper included pre-licensure nurses and other post-licensure healthcare professionals. The inclusion of representatives from more than one profession in the activity was intentional, which is a key component of IPE [22]. Although the students did participate in interprofessional rounds in their clinical experience and had opportunity to interact with students from other health professions, the activities were primarily centered on students from the profession of nursing. As such, we have purposefully called this an introductory IPE activity and view this study as building a foundation for exposure to IPE and future interventional research with a larger sample of multi-disciplinary students.

The introductory IPE activity evaluated in this study was integrated into a BSN program in which the first year is a pre-professional year, taken in the College of Arts and Science. The subsequent 3 years are completed in the College of Nursing, with a focus on core nursing curriculum (a one plus three program). Thus, exposure to multi-professional education begins in the second year of the program, at which time the students experience the first year of the core nursing curriculum. Students naturally experience some learning side-by-side with other healthcare professionals (pre- and post-licensure) during their brief, introductory clinical placements. In the third year of the BSN program (second year of core nursing curriculum), nurse educators begin to explicitly expose nursing students to IPE and IPC principles in both the pediatric theory and clinical courses. The introductory activity evaluated in this paper occurred in the third year of the program; as such, it was intended to provide a supportive foundation for future immersion in interprofessional learning. Thus, this introductory IPE activity was seen as a preliminary and intentional step towards exposing the nursing students to practice collaboratively within and across their discipline in future pre-licensure experiences during their nursing education [25].

This project builds on the Co-Principal Investigators’ previous research [30]. In 2009, the College of Nursing [CofN] at the University of Saskatchewan developed a partnership with other health science Colleges to create introductory IPE learning experiences within the pediatric nursing theory course. These IPE experiences intended to provide nursing students with learning opportunities to begin to develop IPC competencies to be implemented in the pediatric setting. Faculty from the Colleges of Nursing and Dentistry initially collaborated to design a lecture about pediatric oral health for nursing students including topics such as screening and conducting assessments, primary prevention, early care services, and referrals within an interprofessional team, delivered by a pediatric public health dentist. In addition, a case study was collaboratively designed to support students in learning and applying the IPC competencies outlined in the National Interprofessional Competency Framework [25].

Findings from a pilot project indicated a statistically significant (*p* < .001) increase in nursing students’ oral health knowledge from pre-test (67%) to post-test (86%), following this activity. These results, along with the professional expertise and enthusiasm experienced during the development and implementation of the oral health case study led to an expansion into other areas of pediatric content. Four additional interprofessional case studies were added to the third-year pediatric theory course in the CofN. Specifically, the team expanded to include nursing faculty, a perfusionist, pediatric endocrinologist, pediatric palliative care specialist, pediatric oncologist, and pediatric public health dentist.

The introductory IPE activity included in a pediatric nursing theory and clinical course that was evaluated in this study had the following components: 1) an overview of the IPC competencies outlined in the National Interprofessional Competency Framework [25] by nursing faculty; 2) two-hour lectures from a perfusionist, pediatric endocrinologist, pediatric palliative care specialist, pediatric oncologist, and pediatric public health dentist; 3) case studies created collaboratively by the interprofessional team of faculty, provided to the nursing students prior to each lecture for discussion during the class; and, 4) in their related clinical course, students were provided opportunities to come together with other healthcare professional students during daily interprofessional rounds to learn more about other professionals, laying the ground work for future collaborations [25]. To our knowledge, this educational IPE activity is an unique blend of pre-licensure nurses and varying post-licensure healthcare professionals in a nursing educational environment. It was developed by an interprofessional team using the National Interprofessional Competency Framework and evaluated based on its’ ability to improve such competencies. As such, findings from this study add valuable knowledge about innovative ways to create and integrate IPE early in nursing education programs that supports the development of IPC in nursing curricula with the potential for adaptation to other health science faculties and courses.

## Methods and procedures

Ethical approval was obtained from the University of Saskatchewan Behavioural Research Ethics Board (REB) and Operational Approval was provided by the College of Nursing at the university. All methods described herein were performed in accordance with the relevant guidelines and regulations of the University REB. For example, all eligible nursing students were informed of the purpose and procedures of the study by a research assistant (RA) and invited to participate in the study through an informed consent process. Those who decided to participate provided basic demographic information and then completed the ICCAS [26]. Additional details about the process follow.

### Sample

The purposeful sample included third-year nursing students enrolled in the pediatric nursing theory course that included the introductory IPE activity at the CofN at the University of Saskatchewan. The BSN program at the CofN consists of a pre-professional year followed by three years of nursing-specific education. The third-year pediatric nursing theory course involves approximately 26 hours of instruction and is taken alongside a clinical course comprised of 78 hours of clinical practice in pediatric school and acute care settings.

### The introductory IPE activity

The introductory IPE activity was integrated into the pediatric nursing theory course, and included two-hour interactive lectures delivered by a perfusionist, pediatric endocrinologist, pediatric palliative care specialist, pediatric oncologist, and pediatric public health dentist, as well as written case studies relevant to each specialty, completed independently by students before each of the lectures. To begin, the guest lecturers provided a safe learning environment by: having the students complete the case study prior to each class to build confidence in participation; acknowledging that all discussion and responses were appreciated and respected; providing positive and encouraging feedback to all responses; and, using teaching and learning tools such as Mentimeter and KAHOOT! to engage all students including those who may not feel comfortable speaking in class. Together, the nursing students and the guest lecturers reviewed the case studies that the nursing students had completed during each class, discussing questions that arose and confirming correct responses to the questions in the case studies. Two nursing faculty members (Co-Principal Investigators) met with each of the interprofessional guest lecturers multiple times to co-develop IPE content for interactive lectures that included multiple activities and case studies. The objective of this introductory IPE activity was to prepare nursing students for IPC; as such, the development of related materials was guided by the six competencies essential for IPC (*role clarification*, *patient/client/family/community-centered care*, *team functioning*, *collaborative leadership*, *interprofessional conflict resolution*, and *interprofessional communication*) outlined in the National Interprofessional Competency Framework [25].

Examples of how the competencies outlined by the Canadian Interprofessional Health Collaborative [CIHC] [25] were embedded in the interactive lectures and case studies follow. To support *role clarification*, each guest lecturer clearly communicated their role, unique knowledge, and contributions to the interprofessional team. The nursing students were then asked to reflect on the role of nursing in the related interprofessional team and engaged in a discussion around the diversity of the roles and responsibilities of such team members. Each guest lecturer integrated clinical examples of *patient/client/family community-centered care* where they listened to patients and families and viewed them as experts in their plan of care. The four principles outlined by the Institute for Patient- and Family- Centered Care [32], respect and dignity, information sharing, collaboration, and participation, were also integrated into the course to support this competency. *Team functioning, collaborative leadership, and interprofessional conflict resolution* were developed through the interprofessional case studies, which students were given prior to the lectures, and then asked to discuss and collaboratively make decisions about, during the lecture. Clear expectations were set at the beginning of each lecture in relation to listening, negotiating, demonstrating respect, and interacting in a professional manner to develop students’ *interprofessional communication*.

### Data collection

Study data were collected at the end of class time on the final day of the pediatric nursing theory course. An RA explained the purpose of the study and obtained written informed consent for those who agreed to participate. Any student that didn’t want to participate in the study left the class. Participants were asked to complete a basic demographic form (age, gender, marital status, and occupation). Quantitative data were then collected via the ICCAS, which was used to evaluate nursing students’ change in competencies for IPC. The ICCAS is a valid and reliable [27, 33, 34], structured, self-administered, paper-based, retrospective pre- and post-activity survey, which includes 20 items ranked on a 7-point (Strongly Disagree-Strongly Agree) scale [27]. As the ICCAS is a pre-and-post survey, the students were asked to complete the survey after the IPE activities were done by rating their abilities twice: Once as they recalled their abilities before the introductory IPE activities; and, again once all of the IPE activities were done. The 20 items included in the ICCAS make up six subscales that align with the National Interprofessional Competency Framework: interprofessional *communication*, *collaborative leadership*, *role clarification*, *patient/family-centred care*, interprofessional *conflict resolution*, and *team functioning* [27] and was, therefore, appropriate for this study. Furthermore, the National Interprofessional Competency Framework provided an integrated approach to describe the competencies required, and highlight the knowledge, skills, attitudes, and values that shape the judgements essential for effective IPC [25]. Study participants completed the ICCAS [26] on the final day of the pediatric nursing theory course. All study data were de-identified to maintain participant confidentiality.

### Data analysis

Frequencies, percentages, and means were conducted for demographic questions. Cronbach’s alpha coefficient was used to investigate the internal reliability of the ICCAS subscales. All subscales had alpha values greater than .70, indicating high internal consistency. Values are reported in Table 1.

**Table 1:** Cronbach’s alpha for subscales at pre and post-survey

Subscale	Pre	Post
Communication	0.84	0.86
Collaboration	0.85	0.83
Roles and Responsibilities	0.79	0.78
Collaborative Patient/Family-Centered Approach	0.89	0.86
Conflict Management/Resolution	0.76	0.86
Team Functioning	0.86	0.78

Paired samples t-tests were conducted to measure the difference from pre- to post-participation for all 20 items and 6 subscales. Effect sizes (Cohen’s d) were calculated as a measure of practical significance where .2 is small, .5 is medium, and .8 is large. A Bonferroni correction resulted in a revised significance value of *p* = .008. Additionally, confidence intervals (CI) were included to display the probability that a parameter will fall between a pair of values around the mean. Furthermore, the confidence intervals measured the degree of uncertainty or certainty in our sampling method. The CIs in this analysis were constructed using confidence levels of 95%, implying that we are 95% confident in the mean values of the data.

## Results

A total of 180 nursing students completed the introductory IPE activity within the pediatric nursing theory course. Those who agreed to participate in the study (*n* = 111) completed a retrospective pre- and post-IPE activity survey on the last day of the course. The sample had a large representation of women (*n* = 96; 86.5%) versus men (*n* = 15;13.5%), with an age range from 20 to 43 and a mean of 23.96 years (SD = 4.01). Demographic data is presented in Table 2, including participants’ age, gender, ethnicity.

**Table 2:** Demographic Data

(*n* = 111)	
Age in years M (SD)	
Sex	n (%)
Male	11 (13.5)
Female	96 (86.5)
Ethnicity	n (%)
Caucasian	72 (64.9)
Aboriginal	10 (9.0)
South Asian	6 (5.4)
Asian	3 (2.7)
African	4 (3.6)
Hispanic	1 (0.9)
Middle Eastern	1 (0.9)
Multiple ethnicities	2 (1.8)
Canadian	4 (3.6)
Not indicated	8 (7.2)
Occupation	n (%)
Student	110 (99.1)
Not identified	1 (0.1)

The purpose of this study was to examine if exposure to this introductory IPE activity led to a change in the perceived ability of third-year undergraduate nursing students to practice competent IPC. In addressing the purpose and research question, data analysis demonstrated there was a significant difference (*p* < .05) on all items and subscales pre- and post-IPE activity. This indicates that the nursing students perceived an improvement in all competencies following the IPE activity in their pediatric nursing theory and clinical courses. Findings for each subscale are included in Table 3. Effect sizes were large for the subscales and ranged from medium to large on all 20 items.

**Table 3:**
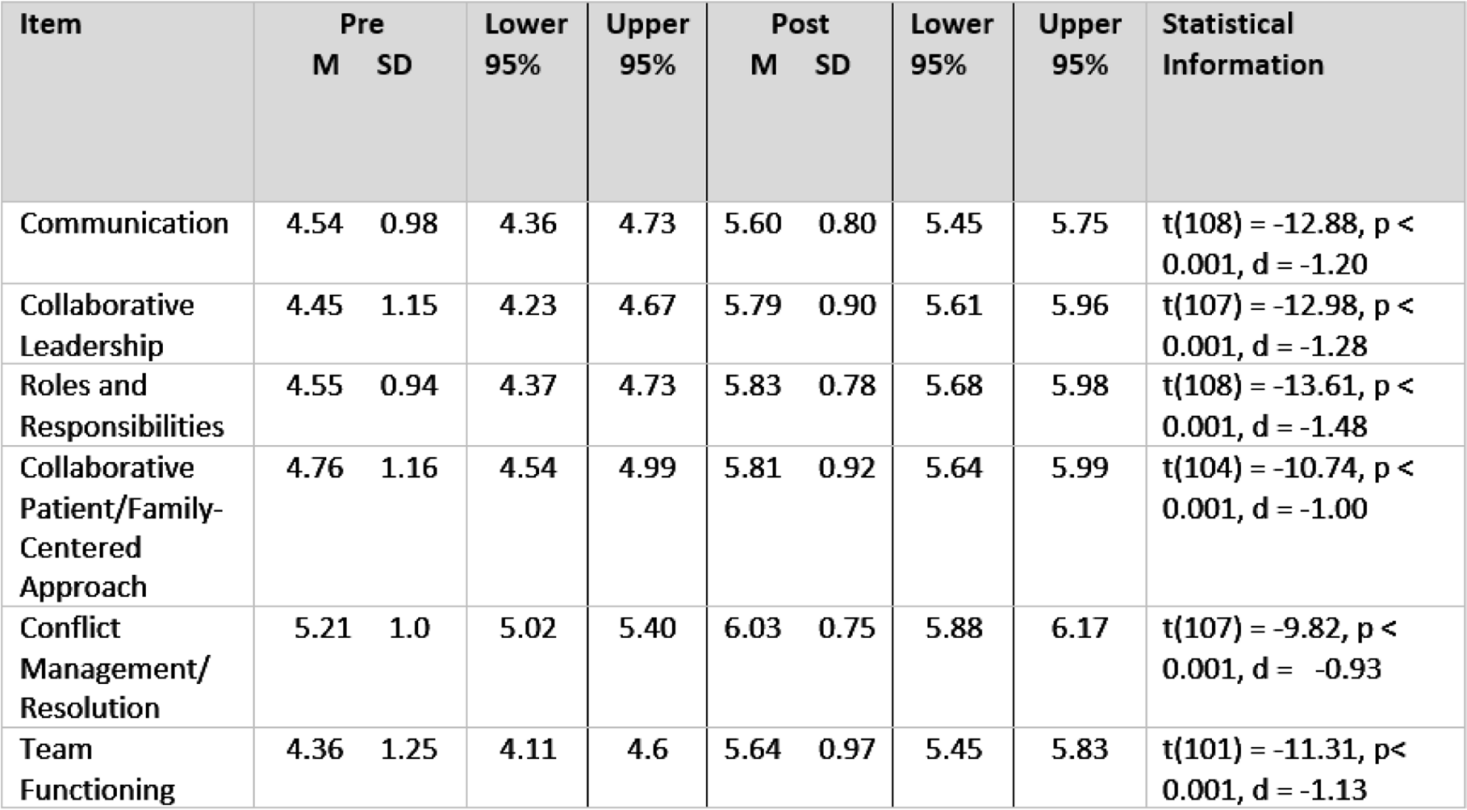
Pre and Post Changes: Means, Standard Deviations and Statistical Information

## Discussion

The introductory IPE activity was used to intentionally integrate all six of the competencies for IPC in a pediatric nursing theory and clinical course. Different roles and responsibilities of nursing and other professions were discussed with each interprofessional lecture. Interprofessional communication, including listening, and developing trust and respect, collaborative leadership, including shared decision-making, and team functioning were developed through small-group discussions and case study activities focused on an interprofessional team approach to patient-and family-centered care. Case study discussions provided nursing students with opportunities to participate in interprofessional problem-solving, as the guest lecturers engaged in discussing the case studies with the students. Questions were specifically designed to create conversation amongst pre-licensure nursing students and post-licensure health professionals, about each of the competencies for IPC. Each nursing student was provided opportunity during their pediatric clinical to participate in interprofessional rounds which allowed the nursing students to interact and learn about their peers from other health professions [28]. Notably, the nursing students’ perceived ability to meet the competencies necessary for IPC improved following the IPE-enhanced pediatric nursing theory course, as demonstrated by significantly higher scores in all competencies (Interprofessional communication, collaborative leadership, role clarification, patient/family-centred care, interprofessional conflict resolution, and team functioning).

### Role clarification

The most significant improvement [t (108) = − 13.61, *p* < 0.001, d = − 1.48] occurred within the *Role Clarification* competency, demonstrating that students’ understanding of their own role and the role of other members of the interprofessional team increasing following the IPE-enhanced pediatric nursing theory and clinical course. Prior to this IPE experience, some of the students discussed their beliefs of professional stereotyping including preconceptions about hierarchy and status which were contributing to a lack of understanding of the role of a physician. Clarke and Greenwald [35] also found that physicians and nurses had inadequate understanding of each other’s role. The existence of professional hierarchies and the lack of understanding of other team member’s roles, both past and present, have the potential to threaten relationships among healthcare professionals and jeopardize the team’s ability to meet the patient/client and family goals [1, 36].

Role Clarification was included in the theory course by each guest lecturer communicating their unique role and the nursing students reflecting on and discussing the roles of different members of the interprofessional team. The nursing students were also exposed to other health care professional students during their clinical experiences providing opportunity to learn more about the role of other professions. The findings of this current study support that Role Clarification is the competency best supported through such an approach. Thus, the results of the current study showed that the nursing students learned about and had improved attitudes towards the role of various health professionals on the team in the pediatric clinical setting. Although these changes in attitudes among nursing students were presented in previous studies [36, 37], the results of the IPE activity confirmed a positive outcome from the IPE learning experiences which included an improved understanding of the roles on an interprofessional team.

### Interprofessional communication and collaborative leadership

The interprofessional competencies, Interprofessional *Communication* [t(108) = − 12.88, *p* < 0.001, d = − 1.20] and *Collaborative Leadership* [t(107) = − 12.98, *p* < 0.001, d = − 1.28] provided evidence of significant learning. Studies have proven that a lack of these competencies among healthcare professionals can result in adverse or sentinel events [1, 38]. Lack of leadership including the inability to communicate during rounds or clinical practice can be detrimental to quality patient- and family- care, and in some cases, leads to fragmented care [4, 35]. These authors argued that some nursing programs have responded by ensuring the inclusion of IPE content into the curriculum including interprofessional communication and leadership skills. One example of a study measuring communication was an interprofessional course offered to academic health science students whereby the researchers found a positive impact on interpersonal and interprofessional communication among the nursing, pharmacy, and medical students [39]. However, data indicate that communication is a competency that is measured the least [40]. The researchers of the present study were strong proponents of IPE and, therefore, decided to intentionally develop and evaluate interprofessional communication and collaborative leadership. Thus, the students were provided opportunities to learn, in both a small group work and as a class, the basic principles of interprofessional communication including listening, developing trust and respect, and shared-decision making. The students were encouraged to express opinions regarding care decisions, and the explicit focus of the communication among students was on a team approach to patient- and family-centered care. In addition to interprofessional communication, in this western Canadian province, leadership skills are a competency for entry to nursing practice; therefore, this IPE activity highlighted that all nurses are leaders and the students were encouraged to practice this competency in this safe pedagogical environment. As recommended by CIHC [25], the collaborative leadership skills activities involved having each student help other team members to stay on task in order to work more effectively together. For example, the students were provided the opportunity to work together and shared decision-making was encouraged in order to complete the questions of the case studies. In addition, during the pediatric cardiovascular lecture, the nursing faculty member and perfusionist showed cinematography of medical pathology taken from live angiograms and supported students in taking the lead on identifying major vessels, congenital cardiac anomalies, and therapeutic interventions when viewing and in classroom discussions. Nursing students were also provided the opportunity to practice their communication and leadership skills alongside students from other health professions in daily care planning in their associated clinical course. The findings of the present study found that the intentional incorporation of these IPE principles into the pediatric courses led to significant and unique learning related to interprofessional communication and collaborative leadership within a pediatric healthcare team. These findings will be helpful to guide additional research aimed at the development, implementation, and/or revisions to undergraduate health professional education programs. Specifically, health professional educators may consider opportunities for students to learn and lead interprofessional communication by organizing activities such as small group work that allow students to practice the basic principles including listening, developing trust, and respect, and shared-decision making.

### Patient/family-centred care

Along with the above-mentioned competencies, *Patient-family-centered care* was a focus of the IPE enhanced course which places family as central; it is working “with” patients and families rather than doing “to” or “for” them [32]. There was a significant difference between the nursing students’ pre-and-post survey responses in this study [t(104) = − 10.74, *p* < 0.001, d = − 1.00]. Although there is a multitude of IPE studies examining the core competencies, few discuss a similar approach of the current study [27, 41]. As recommended by the Institute for Patient- and Family-Centered Care [32], respect and dignity, information sharing, collaboration, and participation were the four principles integrated into the course. For example, in the pediatric palliative care class, the students were given an actual scenario of a 16-year-old and their family who refused pain medication while the adolescent only had weeks to live. The students were first asked to work in groups to develop an approach to the planning, delivery, and evaluation of healthcare that was grounded in a mutually beneficial partnership with the patient/family and pediatric healthcare team [32]. Then, the pediatric palliative care physician, a nursing faculty member, and the larger group of nursing students worked through the scenario using the four principles of patient- and family-centered care to enhance exposure to patient-and-family-centred care. This unique approach to teaching the competency-patient-and-family-centered care resulted in a positive learning outcome for the students.

### Interprofessional conflict resolution

Nursing students’ scores indicated significant improvements in all items of the scale in relation to *Interprofessional Conflict Resolution* following participation in the IPE-enhanced pediatric nursing theory course [t(107) = − 9.82, *p* < 0.001, d = − 0.93]. While this score was the lowest amongst the six competencies, it may be due to the difficulties in enacting conflict resolution in the introductory IPE activity and nursing students’ ability to imagine Interprofessional conflict at this early stage of exposure. However, positive results have also been demonstrated in pharmacy students following IPE simulations that required collaborating with nursing students on patient cases [42], and medicine, dentistry, pharmacy, and optometry students following a day-long case-based introductory IPE experience [43]. In addition, when evaluating an IPE simulation over two consecutive years, King et al. [8] found improvement in conflict management/resolution in the first year.

Interestingly, the Interprofessional Conflict Resolution competency is one that the nursing students rated highest prior to the activity. King et al. [8] found similar results, wherein the competency that nursing students rated the highest prior to the IPE activity was also the one that improved the least. The findings from a recent literature review may support a better understanding of the results of this study and those of King et al. [8]. Specifically, a recent scoping review of ten teaching-learning activities in undergraduate nursing education programs used to inform and develop IPE curricula reported the primary focus of the activities was to advance the competencies interprofessional collaboration, role identification, and team functioning competencies with little to no focus on conflict management [20]. Thus, to our knowledge, few published studies report the activities designed to promote the *Interprofessional Conflict Resolution* competency making these new and unique findings in which the principles were prioritized as important for nursing students.

It is clear that developing confidence to manage conflict is difficult for experienced nurses. Therefore, the *Interprofessional Conflict Resolution* competency was integrated into the case studies with the understanding that it was important to first provide exposure to developing the confidence to manage interprofessional conflict because this can be difficult for novice nurses. The research team developed each of the cases knowing that the students would only be beginning to adapt the ability to address conflict in a constructive manner. To accomplish this task, the students were taught the potential of both the positive nature of conflict and strategies to manage conflict. Then, the students were provided the opportunity to work together, and shared decision-making was encouraged in order to complete the questions in each of the cases. The case study approach was used to promote discussion and opportunity for decent in a safe learning environment, and was an effective approach to begin the process of actively engaging the nursing students in learning about the *Interprofessional Conflict Resolution* competency. The findings of the present study indicated that the intentional incorporation of the IPC competencies into the IPE activity led to a significant improvement in all competencies. Based on these results, it may be valuable to focus future research on increasing understanding of students’ perceived abilities related to *Interprofessional Conflict Resolution* prior to creating IPE activities so that interventions can be introduced to specifically target areas of need.

### Team functioning

*Team Functioning* was also a competency that improved among the nursing students [t(101) = − 11.31, *p* < 0.001, d = − 1.13] and was highlighted by having the students participate in, and discuss the case studies in order to learn to share information and enhance development of communication and listening skills. The case study discussions provided an opportunity to engage in uniprofessional as well as interprofessional shared patient-centered problem solving [25]. Although other studies have investigated various interventions related to teamwork and found positive outcomes [8, 20, 44], the current findings are unique and confirm the importance of teamwork in the development of IPC skills and knowledge. Specifically, the innovative IPE activity empowered nursing students with the knowledge, attitude, and motivation necessary to enhance their ability to act within an interprofessional team while providing high quality pediatric care. The results demonstrated that the students understood the group process and the importance of working on a healthcare team to enable more effective collaboration and improve quality care for pediatric patients and their families. To our knowledge, this is the first study in undergraduate nursing education to examine IPE involving a team of multiple health care professionals such as pediatric endocrinologist, pediatric dentist, pediatric oncologist, pediatric palliative care specialist, and a perfusionist to work together and role model team functioning, and to potentially enhance collaborative pediatric nursing education and practice in a nursing theory and clinical course.

Overall, significant improvements were seen in each of the competencies that were examined using the ICCAS which has been demonstrated in nurse practitioner/midwifery, dental, and medical students following involvement in an IPE clinical simulation and case study activity [18], and nursing, nurse practitioner, and dental students following an IPE oral health community program [16]. To our knowledge, the most similar intervention to the activity evaluated in this current study is that by Aleshire et al. [13] who created a course for third-year BSN students that took an interprofessional and systems perspective on major health issues and health care. Course content was delivered by an interprofessional team of healthcare experts and clinicians, and a section of the course focused on children and families [13]. However, there are two key differences in the studies. First, it does not appear that the National Interprofessional Competency Framework [25] was intentionally integrated into the course, although components such as roles and responsibilities, teamwork, and communication are mentioned. Second, Aleshire et al. [13] measured change in nursing students’ attitudes toward interprofessional health care teams, restricting our ability to compare results. Aleshire et al. [13] mentioned the ICCAS being subsequently done for the same course; to our knowledge this data has not been published. The inclusion of the National Interprofessional Competency Framework [24] in the development of the IPE activity and the use of the ICCAS for measuring change in the six relevant competencies for IPC were essential components of this study. As the introductory IPE activity delivered in this study improved nursing students’ abilities in all six competencies, the process used to develop and evaluate this activity may be suitable for the creation and delivery of effective IPE in pediatric nursing courses. In addition, there is potential to adapt this novel introductory IPE activity for use in other health science courses.

## Limitations and strengths

There are limitations associated with the current study. Given that this was a cross-sectional survey, determining the sustainability of the improvements captured is not possible. In particular, it is not possible to comment on whether the improvements seen in this study influenced the nursing students’ IPC in practice. The ICCAS solely relies on self-report data and there is a risk of self-report bias with such a form of evidence. The students may have experienced some exposure to other healthcare professionals during their brief introductory clinical placements in Year 2 which were unattributed to this study. As such, this initial exposure may have affected the students’ “before participating in IPE activity scores” in the retrospective survey. Additionally, the study included a specific IPE activity in a particular nursing program and the sample was largely homogeneous (single, Caucasian, women), limiting the generalizability of results. Lastly, response bias may have occurred as those students who were most interested in IPC may have agreed to participate in the survey, and students’ previous exposure to IPC was not evaluated. As a result, significance of our findings may be slightly inflated. Strengths of our study include the size of the sample (*N* = 111), the incorporation of all six IPC competencies into the IPE activity, and the unique delivery of IPE by various healthcare professionals. Future research may evaluate the effectiveness and feasibility of such an IPE activity in larger and more diverse samples of multi-professional students, across multiple nursing programs, or a longer length of time. A major strength of this research is the development of an introductory IPE activity that can be used as a foundation for future research.

## Conclusion

The findings from this research demonstrate that nursing students experienced an increased ability to participate in IPC in pediatric practice following the IPE activity. Significant learning occurred in all six IPC competencies including role clarification, patient/client/family/community-centered care, team functioning, collaborative leadership, interprofessional conflict resolution, and interprofessional communication [25]. The introductory IPE activity in this study was designed by a team of interprofessional healthcare faculty and delivered in a classroom setting through interactive lectures and case studies. The inclusion of the National Interprofessional Competency Framework in the development of the IPE activity and the use of the ICCAS for measuring change in the six relevant competencies for IPC were essential components of this study. This information may be used to develop future research aimed at refining and evaluating the feasibility and effectiveness of IPE interventions. Such interventions may well enhance nursing education and encourage nurses to engage in IPC in practice and, ultimately, positively influence collaborative nursing care provided to pediatric patients and their families.

## Data Availability

The datasets generated and/or analysed during the current study are not publicly available due to limited consent from participants but are available from the corresponding author on reasonable request.
